# Myeloid-derived suppressor cell: A crucial player in autoimmune diseases

**DOI:** 10.3389/fimmu.2022.1021612

**Published:** 2022-12-09

**Authors:** Dandan Xu, Cheng Li, Yushan Xu, Mingyue Huang, Dawei Cui, Jue Xie

**Affiliations:** ^1^ Department of Blood Transfusion, The First Affiliated Hospital, School of Medicine, Hangzhou, Zhejiang University, China; ^2^ School of Medicine, Southern University of Science and Technology, Shenzhen, China

**Keywords:** myeloid-derived suppressor cells, DNA methylation, histone modification, non-coding RNAs regulation, autoimmune diseases

## Abstract

Myeloid-derived suppressor cells (MDSCs) are identified as a highly heterogeneous group of immature cells derived from bone marrow and play critical immunosuppressive functions in autoimmune diseases. Accumulating evidence indicates that the pathophysiology of autoimmune diseases was closely related to genetic mutations and epigenetic modifications, with the latter more common. Epigenetic modifications, which involve DNA methylation, covalent histone modification, and non-coding RNA-mediated regulation, refer to inheritable and potentially reversible changes in DNA and chromatin that regulate gene expression without altering the DNA sequence. Recently, numerous reports have shown that epigenetic modifications in MDSCs play important roles in the differentiation and development of MDSCs and their suppressive functions. The molecular mechanisms of differentiation and development of MDSCs and their regulatory roles in the initiation and progression of autoimmune diseases have been extensively studied, but the exact function of MDSCs remains controversial. Therefore, the biological and epigenetic regulation of MDSCs in autoimmune diseases still needs to be further characterized. This review provides a detailed summary of the current research on the regulatory roles of DNA methylation, histone modifications, and non-coding RNAs in the development and immunosuppressive activity of MDSCs, and further summarizes the distinct role of MDSCs in the pathogenesis of autoimmune diseases, in order to provide help for the diagnosis and treatment of diseases from the perspective of epigenetic regulation of MDSCs.

## Introduction

Numerous diseases concerning chronic inflammation, autoimmunity, and different types of cancers can lead to abnormal and persistent accumulation of myeloid cells that deviate from the standard track of differentiation (1). Compared with physiologically differentiated myeloid cells, these mesenchymal stem cells have distinct characteristics, such as immature phenotype and morphology, relatively weak phagocytic function, as well as immunosuppressive function. Based on their function and myeloid origin, this heterogeneous population of myeloid cells is now collectively referred to as myeloid-derived suppressor cells (MDSCs) (2). Initially, studies on MDSCs mainly focused on tumor-bearing mice or cancer patients to study their regulatory mechanisms in tumor pathogenesis. Meanwhile, therapeutic strategies targeting MDSCs have proven to be a well-tolerated and hugely promising therapeutic approach in cancer therapy (1–3). Autoimmune diseases are characterized by the loss of immunological tolerance to self-antigens, leading to the overproduction of autoreactive immune cells and/or autoantibodies and self-tissue damage ultimately (4, 5). However, the pathological mechanism of autoimmune diseases has not been adequately studied (6). Recently, MDSCs have been frequently studied for its immunomodulatory role and identified their therapeutic potential in multiple autoimmune diseases, such as multiple sclerosis (MS), rheumatoid arthritis (RA), systemic lupus erythematosus (SLE) and inflammatory bowel disease (IBD), but with limited clinical application caused by poor understanding of the mechanism (7–10). Meanwhile, there are conflicting findings on the regulatory mechanisms of MDSCs in autoimmune diseases. Some studies believe that MDSCs can alleviate disease because of its anti-inflammatory function. In contrast, others believe that MDSCs have pro-inflammatory and disease-promoting effects in the inflammatory state *in vivo* (11). Therefore, it is quite necessary to identify the regulatory role of MDSCs in the pathogenesis of autoimmune disorders.

Epigenetic modifications refer to reversible and heritable alterations occurring in genomic DNA but do not change the DNA sequence, mainly including DNA methylation, histone modification, and non-coding RNA regulation (7). Epigenetic mechanisms play a significant regulatory role in mediating gene expression that affects the differentiation and development of immune cells (4). Epigenetic modifications combined with transcriptional factors also participate in the development of MDSCs and affect their immunosuppressive functions (12, 13). Meanwhile, gene dysregulation caused by epigenetic changes can lead to a variety of pathological conditions such as autoimmune/inflammatory disorders and/or cancers (14–16). How epigenetic modifications affect the occurrence and progression of autoimmune diseases has attracted researchers’ attention to widely explore (17–19).

In the review, we not only summarize the characteristic and immunomodulatory function of MDSCs, but also discuss the effects of epigenetic modifications on the development and function of MDSCs and their roles in the progression of autoimmune diseases, aiming to further provide novel insights into the treatment for autoimmune disorders.

## Characteristic of MDSCs

MDSCs originate from hematopoietic stem cells (HSCs) as a result of altered myelopoiesis (20–22). At steady-state, bone marrow-derived hematopoietic stem cells can differentiate into immature myeloid cells (IMCs) and eventually into mature monocytes and granulocytes. Various pathological conditions, such as infection or tissue damage, can initiate emergency hematopoiesis to protect the host from pathogenic factors. Under these conditions, myeloid cells are rapidly mobilized from the bone marrow (BM) and activated in response to pathogenic signals such as toll-like receptor (TLR) ligands, damage-associated molecular patterns (DAMPs), and pathogen-associated molecular patterns (PAMPs), causing the upregulation of various inflammatory cytokines. This transient myelopoiesis is terminated after the stimulus is removed, and myeloid cell homeostasis is then reestablished. However, some pathological conditions, such as chronic inflammation, autoimmune diseases and tumor, may cause abnormal and persistent myelopoiesis to deter widespread tissue damage in the host due to unresolved inflammation. Under these conditions, IMCs deviate from normal differentiation and become pathologically activated. Compared with physiologically differentiated myeloid cells, these mesenchymal stem cells have distinct characteristics, such as immature phenotype and morphology, relatively weak phagocytic function, as well as immunosuppressive function. Based on their function and myeloid origin, this heterogeneous population of cells is now collectively referred to as MDSCs (23–25). Depending on the origin and anatomical location, MDSCs are divided into different subsets in both humans and mice (**Table 1**). According to the morphological and phenotypic similarity to monocytes or neutrophils, MDSCs were originally allocated into monocytic MDSCs (M-MDSCs) and polymorphonuclear or granulocytic MDSCs (PMN-MDSCs or G-MDSCs) respectively (26). M-MDSCs in mice are characterized by the expression of CD11b^+^Gr-1^+^Ly6C^hi^Ly6G^-^ and PMN-MDSCs are featured with the expression of CD11b^+^Gr-1^+^Ly6C^-^Ly6G^hi^on their cell surfaces (27). Furthermore, there is a novelty identified subset of mice MDSCs named early-staged MDSCs (e-MDSCs), and the phenotype is CD11b^+^Gr1^+^CCR2^+^Sca1^+^CD31^+^ or CD11b^+^Gr-1^-^MHC-II^-^F4/80^-^ (28, 29). In addition, certain molecules, such as CD49, CD115 CD16l, CD124 and CD31, were also used as surface markers to identify the functional properties of different MDSCs subtypes (30–32). The phenotype of MDSCs is more diverse in different diseases in human than that in mice. In human, there are three mainly subpopulations of MDSCs according to the expression of various surface markers: CD33^+^HLA-DR^−/low^ Lin^-^ is defined as the phenotype of early-staged or immature MDSCs (e-MDSCs/i-MDSCs), containing myeloid progenitors that are not yet mature (28); The phenotype of CD33^+^HLA-DR^−/low^CD14^+^CD15^−^ represents inhibitory monocytes, namely monocytic MDSCs (M-MDSCs); The phenotype of CD33^+^HLA-DR^−/low^CD14^−^CD15^+^/CD66b^+^ represents PMN-MDSCs, which is phenotypically distinct from mature neutrophils and possesses powerful immunosuppressive activity (20, 26, 27). In addition, a novel subpopulation of MDSCs has been identified and defined as fibrocytoid MDSCs (F-MDSCs) with the phenotype of CD11b^low^CD11c^low^CD33^+^ IL-4Rα^+^ in cord blood or peripheral blood of patients with metastatic pediatric sarcoma (32). Although F-MDSCs show powerful immunosuppressive function by producing indoleamine 2, 3 dioxygenase (IDO) to induce the differentiation of regulatory T cells (Treg), but this subpopulation has not been identified in mouse tumor models or in patients with other tumors, and further studies are needed (32, 33). Furthermore, other markers, such as S100A9, CD84, and CD49, that are not specifically expressed on MDSCs have also been used as surface markers to identify MDSCs (34–36). Recently, lectin-type oxidized LDL receptor 1 (LOX-1) and lysosomal-associated membrane protein 2 (LAMP-2) have been specifically detected and served as a novel marker molecule in PMN-MDSCs to identify these cells in the peripheral blood of cancer patients, while further confirmation is needed for a unifying concept in mouse (37).

**Table 1:** Classification and phenotype of MDSCs.

Characteristic	M-MDSCs	PMN-MDSCs	e-MDSCs	F-MDSCs
Origin	IMC and monocytic precursors	IMC granulocytic precursors monocytic-like precursors	IMC	Fibroblasts
Characteristic phenotype in mice	CD11b^+^Gr-1^+^ Ly6C^hi^Ly6G^-^	CD11b ^+^ Gr-1 ^+^ Ly6C^-^ Ly6G^hi^	CD11b^+^Gr1^+^CCR2^+^ Sca1^+^CD31^+^ / CD11b^+^Gr-1^-^ MHC-II^-^F4/80^-^	–
Characteristic phenotype in humans	CD33^+^HLA-DR^−/low^ CD14^+^CD15^−^	CD33^+^HLA-DR^−/low^ CD14^−^CD15^+^/CD66b^+^	CD33^+^HLA-DR^−/low^Lin^-^	CD11b^low^CD11c^low^ CD33^+^IL-4Rα^+^
Novel markers in mice	CD49, CD115 CD16l, CD124, CD31		–	–
Novel markers in humans	S100A9, CD84, LOX-1, PD-1		–	–
Main regulators of suppressive functions	iNOS↑↑↑, ROS↑, Arg-1↑; An antigen non-specific manner	ROS↑↑↑, iNOS↑, Arg-1↑; An antigen-specific manner	Exerte more potent immunosuppressive capacity	Indoleamine 2,3-dioxygenase (IDO) pathway

MDSCs, Myeloid-derived suppressor cells; M-MDSCs, monocytic MDSCs; PMN-MDSCs, polymorphonuclear MDSCs; e-MDSCs, early-stage MDSCs; F-MDSCs, fibrocystic MDSCs; IMC, immature myeloid cells; ROS, reactive oxygen species; iNOS, inducible nitric oxide synthase; Arg-1,arginase 1; ↑↑↑, greatly increased in quantity and activity; ↑, increased in quantity and activity.

## Immunomodulatory functions of MDSCs

**Figure 1:**
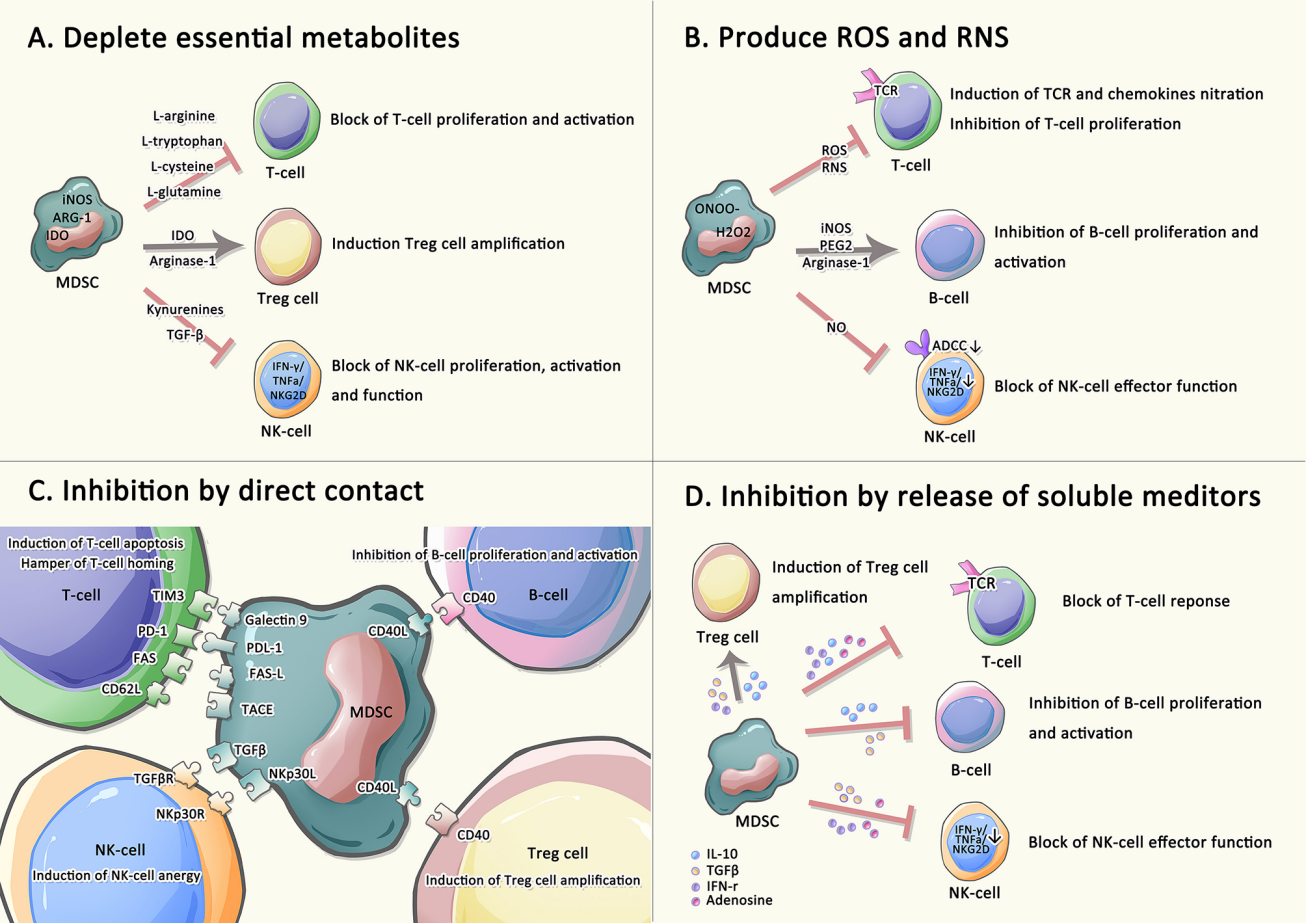
Immune regulatory functions of MDSCs on adaptive immune. Immune effector cells are suppressed by MDSCs undergoing the four main strategies: **(A)** MDSCs depletes amino acids needed for T lymphocyte metabolism. **(B)** MDSCs generate ROS and RNS. **(C)** MDSCs suppress T cells, NK cells, and B cells, as well as induce Treg cells amplification by direct contact. **(D)** MDSCs involve in releasing numerous soluble factors to induce immunosuppression.

Multiple different signals are required for the expansion and activation of MDSCs (6, 38). For instance, granulocyte/macrophage colony-stimulating factor (GM-CSF), IL-6, IL-1ß and prostaglandin E2 (PGE2) are involved in the expansion of MDSCs. IL-13, IL-4, IFN-γ and vascular endothelial growth factor (VEGF) are associated with the activation of MDSCs (39, 40). Through trigger different signal transducers in MDSCs, such as signal transducers and activators of transcription (STATs), nuclear factor kappa-B (NF-κB), and CCAAT enhancer-binding protein beta (C/EBPβ) (17), these factors either induce the expansion of MDSCs and inhibit the differentiation of mature myeloid cells, or directly activate MDSCs and maintain the survival of MDSCs, thereby participating in the regulation of MDSC differentiation, proliferation and apoptosis during hematopoiesis. Many studies have disclosed that MDSCs join distinct immune cells like T cells, B cells, and natural killer (NK) cells to participate in various immune responses and induce immune tolerance (6, 20). Therefore, MDSCs exploit the following four main mechanisms to exert immunomodulatory effectors on immune effector cells: *i*. MDSCs deplete amino acids needed for T lymphocyte metabolism, such as L-arginine, thus inducing T cells proliferation stagnation. Promoted by highly active arginase-1 secreted by MDSCs, L-arginine depletion induces the loss of the CD3ζ chain, thus causing T cells proliferation stagnation and altering T cells immune response. MDSCs promote regulatory T (Treg) cells differentiation and indirectly inhibit the immune response under the synergistic effect of IDO, a rate-limiting enzyme in tryptophan metabolism. Kynurenine, a product of L-tryptophan catabolized by IDO, can prevent NK cells from activation and proliferation, and further restrain their functions. *ii*. MDSCs generate ROS and RNS. NADPH oxidase in MDSC is the main source of ROS, which transfer electrons from NADPH to oxygen to produce superoxide radicals. Accumulated ROS reduces cytokine secretion from T cells. through disrupting CD3ζ chains. The release of RNS also restrains the recruitment and proliferation of T cells through nitration/nitrosylation of TCR and chemokines. MDSCs directly repress the proliferation and activation of B cells through multiple mechanisms such as prostaglandin E2 (PGE2), inducible nitric oxide synthase (iNOS), and arginase. For NK cells, MDSCs can suppress their immune effect functions by releasing NO to inhibit the FC-receptor-mediated antibody-dependent cell-medicated cytotoxicity (ADCC). *iii*. MDSCs exert immunomodulatory effects through direct contact. MDSCs induce T cells apoptosis by expressing Galectin 9, PD-L1 and FAS-L that binds to corresponding receptors on the surface of T cells. Moreover, MDSCs hamper naïve T cells homing through CD62L-TACE interaction and promote NK cells anergy through TGF-β-NKp30L interaction. MDSCs induce Treg cells amplification and inhibit B cells proliferation through CD40-CD40L interactions. *iv*: MDSCs affect the release of soluble mediators. High levels of adenosine not only affect NK cells maturation but also NK and T-cells effector functions. In addition, by releasing IFN-γ, TGF-β and IL-10, MDSCs not only restrain the proliferation and cytotoxicity of T cells but also induce Treg cells amplification, and reduce IFN-γ, TNF-α, and GRZ released by NK cells (21, 40–45) (**Figure 1**). In addition, as different subsets of MDSCs have unique phenotypic characteristics, the mechanism to regulate immune response is also distinct. Many previous studies shown that e-MDSCs have a unique immature phenotype and potent immunosuppressive capacity leading to a crucial role in promoting tumor progression. Nonetheless, the underlying mechanisms of how e-MDSCs regulate cancer and autoimmunity remain unclear (**Table 1**) (29).M-MDSCs mainly mediate inflammatory response by secreting relatively high iNOS and relatively low ROS, while PMN-MDSCs primarily regulate tumors by secreting relatively high ROS and relatively low levels of iNOS (20). M-MDSCs mainly inhibit immune responses in an antigen-non-specific manner, while PMN-MDSCs mainly repress immune responses in an antigen-specific manner, and possess more powerful immunosuppressive functions (46). In addition, F-MDSCs are hypothesized to induce expansion of Treg cells and M2 macrophage populations through IDO pathway (47).

## Epigenetic modification on MDSCs

Epigenetic modification mainly includes DNA methylation, histone modification, and non-coding RNA regulation. As is known, MDSCs are a heterogeneous population of semi-differentiated rather than terminally differentiated immature myeloid cells derived from the bone marrow. Therefore, it gives us a slight clue about how epigenetic reprogramming on MDSCs causes the alteration of its features. Recently, emerging researches have discovered that epigenetic modification may involve in the regulation of MDSCs differentiation, expansion, and immunosuppressive activity. Hence, in this section, we will conclude from recent researches of the role of DNA methylation, histone acetylation, mircoRNA and long non-coding RNA (lncRNA) regulation in the development and immunosuppressive functions of MDSCs (**Table 2**), and therefore provide a view for further study.

**Table 2:** The epigenetic regulation in the development and function of MDSCs.

Epigenetic modulation	Target gene/pathway	Effect on MDSCs	References
**DNA modifications**			
THC	STAT3/S100A8	Promote differentiation and immunosuppressive activity	(53)
DAC	IRF8/TNFα	Sustain survival and accumulation	(48)
**Histone acetylation**			
TSA	NOS^−^/HO^-^	Promote differentiation/expansion	(49)
HDAC2	Retinoblastoma	Promoted the phenotype conversion	(50)
HDAC11	C/EBPβ	Prevented MDSCs differentiation and function	(51, 52)
**miRNAs regulation**			
miR-9	Runx1/SOCS3	Promote differentiation and immunosuppressive activity	(53) (54)
miR-10a	AMPK	Promote expansion and activation	(55)
miR-21/miR-181	STAT3, C/EBP, MLL1	Promote differentiation and immunosuppressive activity	(56, 57)
miR-34a	N-myc	Inhibit apoptosis	(58, 59)
miRNA-30a	SOCS3	Increases differentiation and immunosuppressive activity	(60, 61)
miR-125	JAK/STAT3	Promote accumulation and immunosuppressive activity	(62)
miR-494	PTEN//PI3K/Akt	Promote accumulation and immunosuppressive activity	(63)
miR-210	Arginase/NO	Enhance immunosuppressive activity	(64)
miR-155	SHIP-1/HIF-1α	Promote expansion and immunosuppressive activity	(65–67)
miR-200C	PTEN/FOG2	Increase immunosuppressive activity	(68)
miR-17 family	AML1	Prevente differentiation and immunosuppressive activity	(69)
miR-146a	IRAK1/TRAF6/NF-kB	Inhibit expansion and function	(70, 71)
miR-223	MEF2C	Suppress accumulation and function	(72–74)
miR-142-3p	C/EBPβ/STAT3	Prevente MDSCs differentiation and immunosuppressive activity	(75)
miR-6991-3p	Galectin 9, STAT3	Prevente expansion and promote apoptosis	(76)
miR-15 family	PD-L1/PD-1	Inhibit suppressive functions	(77, 78)
miR-124	STAT3	Reduce the frequencies of MDSCs	(79)
**LncRNAs regulation**			
Olfr29-ps1	miR-214-3p/MyD88	Promote differentiation and expansion	(80)
RUNXOR	RUNX1, Arg1, iNOS, and STAT3	Promote development and suppressive functions	(81)
MALAT1	Arg-1	Inhibit differentiation	(82)
HOTAIRM1	HOXA1-miR124 aix	Promote development and suppressive functions	(83, 84)
RNCR3	miR-185 CHOP	Increase immunosuppressive activity	(85, 86)
Pvt1	Arg-1, ROS	Increase immunosuppressive activity	(87)
AK036396	Ficolin B	Inhibit differentiation	(88)

THC, 19-tetrahydrocannabinol; DAC, Decitabine; TSA,Trichostatin A; HDAC2, histone deacetylase 2; HDAC11,histone deacetylase 11; STAT3, signal transducer and activator of transcription; IRF8, interferon regulatory factor 8; TNFα, tumor necrosis factor; NOS, nitric oxide synthase; HO, heme oxygenase; C/EBPβ, CCAAT/enhancer-binding protein beta; Runx1, RUNX family transcription factor 1; SOCS3, suppressor of cytokine signaling 3; AMPK, adenosine 5’-monophosphate (AMP)-activated protein kinase; C/EBP, CCAAT/enhancer-binding protein; JAK, janus kinase; PTEN, phosphatase and tensin homologue deleted on chromosome 10; PI3K, phosphatidylinositol 3-kinase; Akt, protein kinase B; SHIP-1, src homology 2-containing jnositol phosphatase-1; HIF-1α, hypoxia-inducible factor-1α; AML1, acute myelocytic leukemia; TRAF, tumor necrosis factor receptor-associated factor; NF-kB, nuclear factor kappa-B; MEF2C, myocyte Enhancer Factor 2C; PD-1, programmed death 1.

### DNA modifications

DNA methylation is a form of DNA chemical modification in which the cytosine 5’ carbon level covalent bond of the genomic CpG dinucleotide binds to a methyl group under the action of DNA methylation transferase (DNMTs) including DNMT1, DNMT3a, DNMT3b, and DNMT3L (89–91). On the contrary, ten-eleven translocation methylcytosine dioxygenase (TET) enzymes including TET1, TET2, and TET3 mediate DNA demethylation and activate transcriptions. The regulatory balance of TETs and DNMTs maintains the promoter’s non-methylation/methylation state (92). Recently, more researches paid attention to the function of DNA methylation at specific sites of individual genes during the activation and differentiation of MDSCs. A research demonstrated a downregulation of DNMT3a but an upregulation of TET enzymes in CD33^+^ HLA-DR^–^ myeloid cells, compared with antigen-presenting cell (APC). Meanwhile, researchers found that CpG islands in the promoter regions of TGF-β1, TIM-3, and ARG1 were highly unmethylated in CD33^+^HLA-DR^–^ cells compared with APCs, indicating that the expression of above three genes was principally regulated through DNA methylation in CD33^+^HLA-DR^–^ myeloid cells (93). In addition, How DNA methylation regulate the development and immunosuppression activity of MDSCs has been sufficiently studied by administering 19-tetrahydrocannabinol (THC), an effective inducer of MDSCs (94). THC-induced MDSCs expressed high levels of S100A8, which is essential for the enhanced suppressive function. Overall, this study revealed that THC mediates epigenetic changes to promote MDSC differentiation and function and that S100A8 plays a critical role in this process (94, 95). Additionally, in tumor-bearing mice, *Tnf and Irf8* promoter DNAs in MDSCs showed hypermethylation, resulting in significantly increased accumulation of MDSCs and significantly decreased activation of antigen-specific cytotoxic T lymphocytes (CTL). Instead, Decitabine (DAC), a DNA methyltransferase inhibitor, dramatically decreased the *Tnf and Irf8* promoter DNA methylation and increased the level of interferon regulatory factor 8 (IRF8) and tumor necrosis factor (TNFα) in MDSCs *in vitro*. Decreased DAC-induced aggregation of MDSCs correlates with increased expression of the myeloid lineage-specific transcription factor IRF8 in MDSCs (48). Additionally, IL-6 is a potent activator of STAT3, which not only induced STAT3 activation but also significantly increased the expression of DNMT1 and DNMT3b. IL-6 treatment also contributed to reduced TNFα production and increased J774M cell proliferation. Hence, autocrine IL-6 triggers the STAT3-DNMT intrinsic signaling pathway, and therefore *Tnf* promoter is hypermethylated, promoting MDSCs survival and accumulation (**Table 2**) (48).

### Histone modifications

Histone modification are a class of post-translational modifications that regulate gene expression, including methylation, acetylation, phosphorylation, adenylylation, ubiquitination (96). The process of acetylation in lysine residues is well studied and regulated by the dynamic balance between histone acetyltransferases (HATs) and histone deacetylases (HDACs), which have opposing functions. Generally, acetylation promotes gene expression while deacetylation causes suppression of gene expression. HDAC inhibition enhances histone acetylation, leading DNA to bind tighter and gene expression to reduce (96, 97). Several studies have illustrated the regulatory effects of different types of HDAC inhibitors on MDSCs expansion and function through distinct signal pathways (98). Trichostatin A (TSA), a potent histone deacetylatase (HDAC) inhibitor, induces GM-CSF-mediated bone marrow cells to differentiate into M-MDSC *in vitro*, and this MDSCs have powerful activity in repressing the proliferation of CD4^+^ T cells through nitric oxide synthase (NOS)^−^ and heme oxygenase (HO)^-^ dependent manner (49). HDAC2 facilitates the conversion from M-MDSCs to PMN-MDSCs as it directly binds to retinoblastoma gene (rb1) promoter and causes silencing of rb1 expression in cancer (50). A recently identified member of the HDAC family named HDAC11 mediates expression of C/EBPβ and some other immunosuppressive molecules in MDSCs (51, 52). Jie Chen et al. reported that if without HDAC11, the arginase level and enzymatic activity would be significantly higher in the tumor-infiltrated PMN-MDSCs, whereas iNOS expression and NO production were observed to be significantly higher in the tumor-infiltrated M-MDSCs compared with wild-type (WT) controls. Subsequently, the research further demonstrated that in MDSCs lacking HDAC11, the elevated expression of immunosuppressive molecules was associated with the up-regulation of C/EBPβ. Remarkably, further studies found that the expression of C/EBPβ was highest in CD11b^+^ Gr-1^+^ MDSCs isolated from tumor-bearing mice. Hence, HDAC11 impacts immunosuppressive molecules’ expression in MDSCs through manipulating C/EBPβ expression (**Table 2**) (52).

### Non-coding RNAs regulation

#### miRNA regulation

miRNAs are short interfering non-coding RNAs that regulate post-transcriptional and post-translational gene expression by binding to the 3′ untranslated regions (3′UTR) of target mRNAs (99–101). Numerous miRNAs were considered important in the epiregulation of many immune cells such as DCs, B and T lymphocytes and macrophages (102). Furthermore, emerging literature suggests that miRNAs are vitally related to the differentiation, development, and function of MDSCs (**Table 2**) (77).

A great many miRNAs display a positive regulatory influence on the development and immunosuppression role of MDSCs. miR-9 inhibits the development and promotes the immunosuppressive activity of MDSCs *via* specific binding to the Runt-related transcription factor 1 (Runx1) (53). miR-21 and miR-181b promotes MDSCs generation by targeting STAT3 and C/EBP in sepsis-induced inflammatory response, while when inhibiting these miRNAs, the number of MDSCs is reduced and the late-sepsis survival was significantly enhanced (56). In addition, miR-21 as well as miR-181b positively regulate PMN-MDSCs expansion, activation, and differentiation by inhibiting the expression of mixed lineage leukemia-1 (MLL1) complex (57). miR-9 and miR-181a facilitates the development and immunosuppressive function of e-MDSCs through the interference with suppressor of cytokine signaling 3 (SOCS3) and protein inhibitor of activated STAT3 (PIAS3), separately, thus leading to immune escape and tumor growth in breast cancer (54). miR-10a activates AMPK signaling to promote the expansion and activation of MDSCs in breast cancer cells with chemotherapy-induced immune resistance (55). miR-34a reduces the apoptosis of MDSCs by inhibiting N-myc expression (58, 59). miR-30a increases the differentiation and immunosuppressive activity of MDSCs by targeting SOCS3 and stimulating JAK2/STAT3 signaling, thereby reducing CD8^+^ T cells infiltration and accelerating tumor progression (60, 61). miR-125 family, which includes miR-125a-5p, miR-125b-5p, and miR-351-5p, induces the accumulation and immunosuppressive activity of MDSCs *via* triggering JAK/STAT3 pathway (62). miR-494 plays an essential role in governing the accumulation and functions of MDSCs by targeting phosphatase and tensin homolog (PTEN) and PI3K/Akt pathway (63). miR-210, whose expression was dramatically induced by hypoxia-inducible factor 1a (HIF-1α), enhances the immunosuppressive activity of MDSCs by increasing arginase activity and NO production (64). miR-155 promotes the accumulation of functional MDSCs in the tumor microenvironment (TME) *via* suppressing suppressor of cytokine signaling 1 (SOCS1) signaling and reducing the generation of Treg cells (54, 55, 57–67). miR-200c enhances the suppressive activity mediated by MDSCs. This is achieved through regulating PTEN and FOG2 signaling (68).

**Figure 2:**
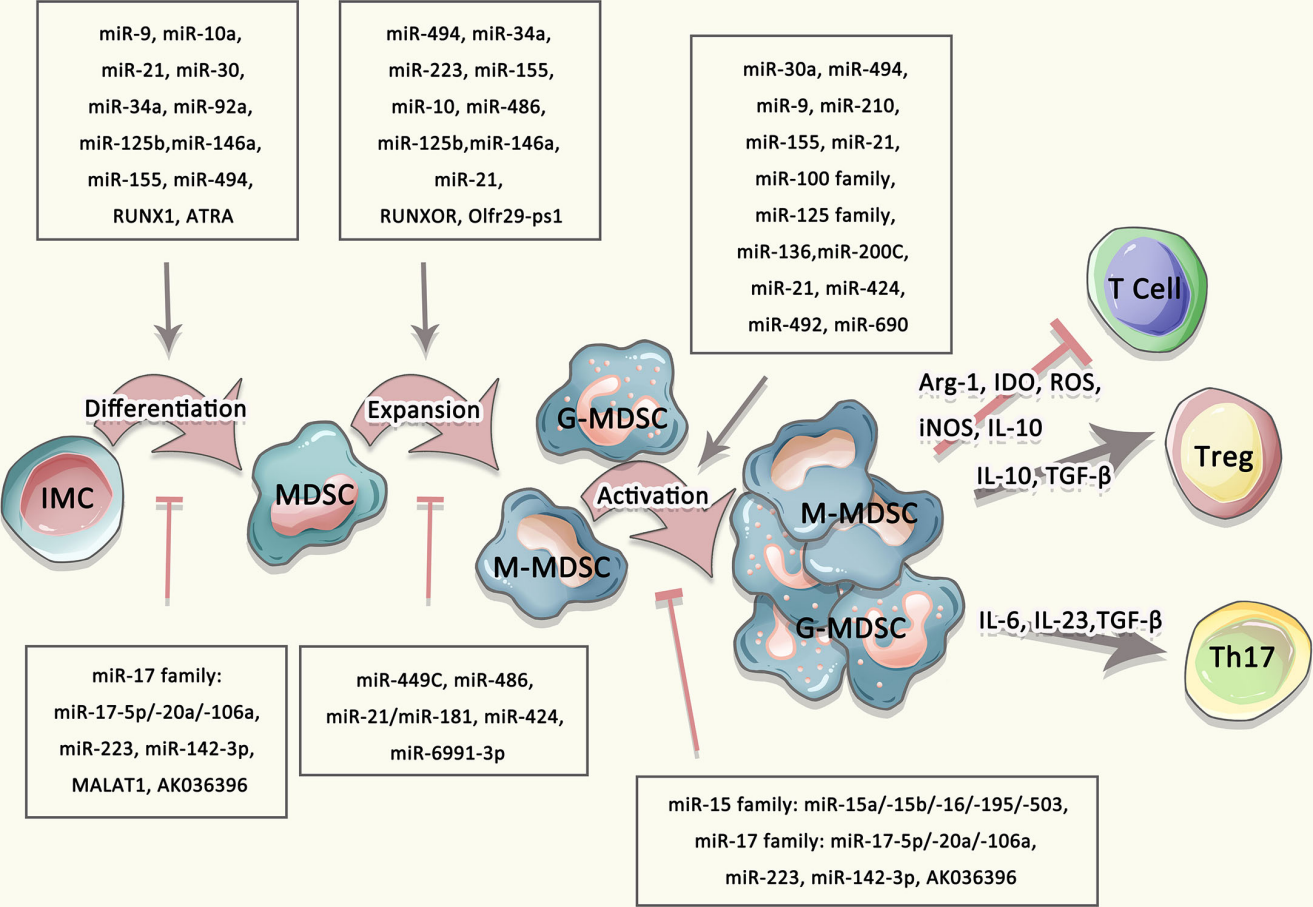
Multiple agents regulated the differentiation, expansion, and activation of MDSCs. The differentiation and expansion of MDSCs were regulated by multiple mediators, such as IL-10, IL-23, TGF-β as well as miRNA, lncRNA.

miRNAs also have a negative contribution in regulating the differentiation and activity of MDSCs. Recent findings have declared that miR-17 family, including miR-17-5p, miR-20a, and miR-106a, are capable of inhibiting tumor MDSCs. miR-17 family overexpressed in human myeloid progenitor cells greatly inhibits AML1 by binding to its AML1 promoter, resulting in down-regulation of M-CSFR, thereby limiting the differentiation of myeloid progenitors into MDSCs (69). Besides, Overexpression of miR-146a inhibits the expansion of MDSCs, thereby delaying tumorigenesis (70, 71). miR-223 significantly inhibits the differentiation and accumulation of MDSCs as well as their suppressive function by targeting myocyte enhancer factor 2C (MEF2C) and inhibiting STAT3 signaling respectively in tumor-bearing mice (72–74). miR-142-3p not only restraints the generation and reduces the immunosuppressive activity of M-MDSCs, but also restores CD8^+^ T cells proliferation through modulating STAT3 and C/EBPβ signal pathways during tumor-induced myelopoiesis (75). Mimic transfection of miR-6991-3p can remarkably inhibit expansion and promote apoptosis of MDSCs by suppressing the activation of LGALS9-mediated JAK and STAT3 signaling pathways (76). miR-15 family blocks the signaling of PD-L1/PD-1, and hence impairs immunosuppression function of MDSCs and/or Treg cells (77, 78). In hepatitis C hosts, miR-124 downregulates the expression of STAT3, IL-10, and TGF-β to reduce the frequency and suppressive function of MDSCs, thereby promoting Treg cells maturity (79). Taken together, a variety of miRNAs are essential to regulating differentiation, maturation and function of MDSCs (**Figure 2**).

#### LncRNA regulation

Long non-coding RNA (lncRNA) is a class of RNA with a transcript length of more than 200 nt, which can regulate the expression level of genes epigenetically, transcriptionally, and post-transcriptionally etc. (103, 104). In addition to miRNAs, lncRNAs are also indicated to involve in the differentiation and development of MDSCs (105). Olfr29-ps1, a lncRNA pseudogene highly expressed in MDSCs, positively regulates the differentiation and function of MDSCs through a regulatory network, which is m6A-modified Olfr29-ps1/miR-214-3p/MyD88 dependent (80). Silencing or ectopic expression of RUNXOR or RUNX1 in CD33^+^ myeloid cells affects MDSCs differentiation and immunosuppressive functions. The overexpression of RUNXOR significantly increases the expression of RUNX1, Arg1, iNOS, and STAT3 at mRNA and protein levels, thus promoting the differentiation of immature myeloid cells into MDSCs during HCV infection (81). Another nuclear intergenic of lncRNA named Metastasis associated lung adenocarcinoma transcript 1 (MALAT1) is also involved in the differentiation of MDSCs cells. Knockdown of MALAT1 significantly increases the quantity of MDSCs by regulating their differentiation in patients with lung cancer (82). HOXA transcript antisense RNA myeloid-specific 1 (HOTAIRM1), preferentially expressed in the myeloid lineage, promotes the differentiation and suppresses function of MDSCs by inducing HOXA1 expression in lung cancer MDSCs to prevent tumor progression (83, 84). RNCR3 stimulates the activity and differentiation of MDSCs by miR-185-5p. In the inflammatory and tumor microenvironment, RNCR3 in MDSCs are expressed significantly higher (85, 86). The increased expression of Pvt1 which is induced by HIF-1α under hypoxia causes the down-regulated expression of Arg-1 and ROS in PMN-MDSCs, and assists T cells to suppress tumor growth in tumor-bearing mice (87). The reduction of lncRNA AK036396 stimulates the development of PMN-MDSCs and inhibits their immunosuppressive function through reducing Fcnb protein stability in a ubiquitin-proteasome dependent manner (88). In summary, numerous current research has emphasized that certain miRNAs/lncRNAs play an important role in the differentiation, development and activity of MDSCs (summarized in **Table 2**).

## Role of MDSCs on autoimmunity disease

The importance of MDSCs in autoimmune diseases is remarkable. If the immunosuppressive activity of MDSCs in cancer and infectious diseases is detrimental to diseases prognosis, the role of MDSCs in autoimmune diseases is undetermined and even controversial. Conflicting studies have shown that MDSCs play a positive and negative role in regulating the progression of autoimmune diseases such as MS, RA, SLE and IBD. In addition, since epigenetic modification of MDSCs is one of the key mechanisms regulating immune tolerance, its regulatory mechanism has also been intensively studied in autoimmune diseases. The changes in differentiation, development and activity of MDSCs induced by epigenetic modifications can reconstruct the immunomodulatory function of MDSCs, which may provide some insights for the harmful or beneficial effects of MDSCs in various pathological conditions.

Hence, in this section, we conclude the immunomodulatory mechanisms of MDSCs in different autoimmune diseases and collected the latest evidences on the epigenetic regulation of MDSCs in the pathogenesis of inflammatory autoimmune diseases from the following four examples, to provide a new perspective for the epigenetic therapies of diseases (summarized in **Tables 3**, **4**).

**Table 3:** The potential role of MDSCs in different autoimmune diseases.

Disease/model	organs	Type of MDSCs	Target gene/pathway	Role of MDSCs	References
MS patient	PB	MDSCs ↑	–	Anti-inflammatory	(106)
EAE	SP, PB, CNS	M-MDSCs ↑	NO, IL-1β	Pro-inflammatory	(107–110)
EAE	SP, PB	PMN-MDSCs ↑	PD-L1/IFN-r	Anti-inflammatory	(111)
RA patient	SF	PMN-MDSCs ↑	–	Ameliorate disease progression	(112)
RA patient	PB	MDSCs ↑	Arg-1, TNF-α, IL-1β	Deteriorate CIA by promoting the expansion of Th17 cells	(113)
CIA	SP	PMN-MDSCs ↑	IFN-r, IL-2, TNF-α, and IL-6; IL-10	Ameliorate CIA by inhibiting Th17 cells	(114, 115)
PGIA	SF	PMN-MDSCs ↑	NO,ROS	Ameliorate PGIA by suppressing DC maturation and expansion of autoreactive T cells	(116)
CIA	SP	MDSCs ↑	Arg-1, TNF-α, IL-1β	Deteriorate CIA by promoting Th17 cells	(117)
CIA	PB	PMN-Exo	PGE2/GSK-3β/CREB	Ameliorate CIA by promoting IL-10+Breg cells production	(118)
SLE patient	PB	M-MDSCs↑	iNOS/Arg-1-dependent manner	Positively correlated with the disease activity	(119–121)
MRL/lpr mice	PB	MDSCs↑	ROS, IL-1β	Deteriorate disease by altering the ratio of Th17 and Treg cells	(122)
Pristane-induced lupus mice	PB, SP	M-MDSCs ↑	cell-cell contact, NO, and PGE2	Ameliorate lupus by inhibitingTh1 cell differentiation and expaning Treg cell.	(123)
Roquinsan/ san SLE mice	SP	MDSCs↑	NO	Ameliorate renal symptom by expansingIL-10^+^Breg cells	(124)
IBD patient	PB	M-MDSCs ↑	–	Positively associate with disease activity	(8)
IBD patient	PB	PMN-MDSCs ↑	CEBPβ	Promote chronic intestinal inflammation by enhancing T cell proliferation	(125)
Hp-induced colitis	SP	MDSCs ↑	I-cysteine/H2S pathway	Alleviate colon inflammation by limiting PMN-MDSCs recruitment	(126)
VILLIN-HA mice	SP, LN	M-MDSCs ↑	NO-dependent, cell-cell contact	Induce T cell apoptosis and suppress T cell proliferation	(127)
DSS-induced colitis	SP	MDSCs ↑	IL-17, TNF, and IFN-r	Ameliorate DSS-induced colitis by adoptive transfer MDSCs	(128)
DSS-induced colitis	SP	PMN-Exo	Arg-1	Ameliorate DSS-induced colitis by preventing Th1 cell proliferation and promoting Treg cell expansion	(129)

EAE, experimental autoimmune encephalomyelitis; MS, multiple sclerosis; RA, rheumatoid arthritis; SLE, systemic lupus erythematosus; IBD, inflammatory bowel disease; CNS, central nervous system; SP, spleen; PB, peripheral blood; LN, lymph node; SC, spinal cord; SF, synovial fluid; BM, bone marrow; NO, nitric oxide; ROS, reactive oxygen species; PMN-Exo, PMN-MDSCs-derived exosomes; PGE2, Prostaglandin E2; GSK-3β, glycogen synthase kinase-3; CREB, cAMP-response element binding protein; CEBPβ, CCAAT/enhancer-binding protein beta. ↑, increased in quantity and activity.

**Table 4:** Epigenetic mechanisms affecting the role of MDSCs in different autoimmune diseases.

Epigeneticmechanisms	Disease/model	Type of MDSCs	Target gene/pathway	Role of MDSCs	References
**Histone modifications**					
EZH2	DSS-induced colitis	MDSCs	STAT3	Ameliorate experimental intestinal inflammation	(130)
**Non-coding RNAs**					
miR-223	MS patient/ EAE	M-MDSCs	ARG1, STAT3	Aggravate EAE severity	(73)
miR-29a-3p miR-93-5p	RA patients and CIA	PMN-Exo	T-bet, STAT3	Ameliorat CIA/RA by inhibiting Th1 and Th17 cell differentiation	(131)
miR-451a	pristane- induced lupus mice	M-MDSCs	IRF-8/miR-451a/ AMPK/mTOR	Aggravate lupus symptoms by regulating M-MDSCs differentiation	(132)
miR-322-5p	SLE	MDSCs	Arg-1/miR-322-5p/TGFβ/SMAD/pathway	Elevat the Th17/Treg ratio and aggravate SLE disease	(133)
LncRNA NEAT1	MRL/lpr mice	PMN-MDSCs	NEAT1-BAFF axis	Aggravate lupus symptoms	(134, 135)

### Multiple Sclerosis

Multiple Sclerosis (MS) is a potentially chronic disabling disease of the central nervous system (brain and spinal cord) (107). The regulatory role of MDSC in MS has been extensively carried out through experimental autoimmune encephalomyelitis (EAE), which is a prevalent and persuasive animal model for MS. In murine models of EAE, circulating CD11b^+^CD62L^+/-^Ly6C^hi^ myeloid precursors were mobilized increasingly and accumulated in the blood, spleen, and CNS. M-MDSCs isolated from the spleen potently induced T cells apoptosis *via* NO production (108–110). Moreover, the above-mentioned MDSCs could prevent the proliferation of T cells and promote the differentiation of IL-1β-mediating inflammatory Th17 cells (136). Hence, the above studies in EAE implied that MDSCs play a pro-inflammatory role and the accumulation of MDSCs was positively correlated with the clinical score/disease severity. However, other studies drew different conclusions. Ioannou et al. found that prior to resolution of inflammation, a large number of CD11b^hi^Ly6G^+^Ly6C^-^ PMN-MDSCs were gathered in the spleen of mice with EAE. Meanwhile, adoptive transfer of PMN-MDSCs inhibited the proliferation of Th1 and Th17-cells through the programmed death ligand (PD-L1)/IFN-r dependent pathway, thereby effectively delaying the progression of EAE (111). In patient diagnosed with MS, the frequency of MDSCs increased in patients with relapsing-remitting multiple sclerosis during relapse compared to patients with stable disease. However, the frequency of MDSCs was reduced in patients with secondary progressive multiple sclerosis (106). Taken together, These observations indicate that MDSCs have multiple functions as organ-specific effectors in MS, with pro-inflammatory functions that aggravate disease progression and anti-inflammatory functions that delay disease progression. Therefore, we speculate that PMN-MDSCs have a protective effect on tissue damage caused by acute inflammation, but M-MDSCs induced in chronic inflammatory environments may play a pathogenic role.

The discrepancy of different studies suggests that it is necessary to further investigate the regulatory role of MDSCs in various autoimmune environments from the perspective of epigenetic modifications (137). Claudia Cantoni et al. observed that patients diagnosed with MS have less circulating suppressive MDSCs with decreased M-MDSCs subset particularly, and the expression of miR-223 was also increased in MDSCs compared with normal controls. miR-223 knockout mice displayed increased accumulation of MDSCs, decreased immune response capacity of T cells isolated from the CNS, and milder disease symptoms. Besides, the up-regulation of Arg-1 and STAT3 are associated with stronger inhibitory function of miR-223^−/−^M-MDSCs. In conclusion, it is indicated that miR-223 shall be pivotal in EAE by mediating the biological behavior of MDSCs and miR-223 can be act as potential and promising target to therapeutic applications (73).

### Rheumatoid arthritis

Rheumatoid arthritis (RA) is a progressive inflammatory autoimmune disease that can lead to destruction in articular cartilage and bone (138). MDSCs play an important role in the pathogenesis of RA, but their effects are still controversial (139, 140). In patients with RA, MDSCs isolated from SF were mainly PMN-MDSCs, which could strongly inhibit the proliferation of autologous T cells and relieve joint inflammation (112). In a mouse model of collagen-induced arthritis (CIA), MDSCs separated from the spleens suppressed the production of pro-inflammatory cytokine, whereas promoted the production of IL-10. Further studies have found that these MDSCs inhibited the proliferation of CD4^+^T cells and their differentiation into Th17 cells, whereas promoted the expansion of Treg cells *in vitro*, leading to the alleviation of joint inflammation and the reduction of CIA severity. Adoptive transfer of PMN-MDSCs ameliorates disease symptoms (114, 115). In addition, PMN-MDSCs isolated from the synovial fluid in the joints have a potently protective effect *via* suppressing the maturation of DCs and the proliferation of autoreactive T cells in proteoglycan-induced arthritis (PGIA) mice (116). Contrary to the anti-inflammatory effect of MDSCs in RA, MDSCs were significantly amplified in arthritis mice and patients with RA, and were positively correlated with disease severity and inflammatory Th17 cells response (113, 117). In a mouse model of CIA, the frequency of MDSCs in the spleen was increased, which could secrete higher level of Arg-1, TNF-α, IL-1β and induce Th17 cells differentiation compared to the control group. Adoptive transfer of MDSCs separated from spleen of CIA mice accelerated the pathogenesis of the disease. Furthermore, the frequency and activity of MDSCs were increased in the peripheral blood of RA patients and associated with increased Th17 cells and disease activity. These results suggested that MDSC promoted Th17 cells differentiation in an IL-1β-dependent manner, thus exerting crucial role in the pathogenesis of RA (113).

Although a growing body of evidence highlighted the important role of disrupted epigenetic regulation of immune cells in the pathogenesis of RA, current research clues regarding the regulatory role of epigenetic modifications in MDSCs in the pathological progression of RA are still weak. PMN-MDSCs derived exosomes (PMN-Exo), playing a similar role as MDSCs, efficiently mitigated the mean arthritis index, joint destruction and leukocyte infiltration in CIA mice. miR-29a-3p and miR-93-5p contained jn PMN-Exo suppressed the differentiation of Th1 and Th17-cells *via* specifically binding to T-bet and STAT3 respectively. Further study indicated that human PMN-Exo exhibited high levels of miR-29a-3p and miR-93-5p as well as powerful ability to inhibit Th1 and Th17-cells differentiation *in vitro*, while M-MDSCs exosomes have no such ability (131).In addition, Prostaglandin E2 (PGE2) in PMN-Exo promoted the production of IL-10^+^Breg cells by activating the signaling pathways of GSK-3β/CREB in CIA mice, thereby alleviating arthritis (118).

### Systemic lupus erythematosus

Systemic lupus erythematosus (SLE) is a life-threatening autoimmune disease characterized by the immune system attacking its own tissues, causing widespread inflammation and tissue damage in the affected organs (141). Although current studies show that MDSCs play an important role in SLE, their immunoregulation effect remains controversial, which may be related to the heterogeneity of MDSCs themselves and the immune microenvironment at different stages of the disease. The frequency of MDSCs in the peripheral blood of active SLE patients were significantly increased compared with normal controls (119). Further studies have revealed that in newly diagnosed SLE patients, the elevated levels of circulating M-MDSCs are positively correlated with disease severity and exert an immunosuppressive effect in an iNOS-dependent manner (120). Besides, in patients with active SLE and a mouse model of lupus, the enhanced MDSCs induced Th17 cells differentiation in an arginine-dependent manner and altered the ratio of Th17 cells and Treg cells *via* ROS and IL-1β dependent manner, thereby exacerbating disease progression (121). Adoptive transfer of MDSCs into MRL/lpr mice induced elevated serum IgG, anti-dsDNA, IL-17A and IL-1β levels, and increased Th17 cells proportions and decreased Treg cells proportions in the spleen (122). However, MDSCs also perform protective effects in SLE progression. In pristane-induced lupus mice mode, M-MDSCs were markedly increased in spleen and peripheral blood and showed immunosuppressive characteristics. Besides, M-MDSCs strongly impaired Th1 cells proliferation but enhanced Treg cells differentiation in a manner depending on cell-cell contact, NO, and PGE2 (123). In Roquinsan/san SLE mice, MDSCs induced the expansion of regulatory B (Breg) cells *in vitro via* iNOS, thus promoting IL-10 production. Moreover, Treatment with MDSCs caused a decrease of autoreactive B cells and an increase of Breg cells, resulting in a reduction of serum anti-dsDNA antibody levels and degree of proteinuria, thus improving renal pathology (124). All in all, the above studies displayed that both the frequency and immunomodulatory activity of MDSCs have a great impact on the development and severity of SLE.

Notably, epigenetic modifications have as well been well documented as critical factors in the pathogenesis of SLE (142, 143). Research indicated that the increase of M-MDSCs is associated with lupus symptoms in mice with pristane-induced lupus. Further study indicated that compared with control mice, miR-451a promotes MDSC differentiation by targeting IRF-8, which was highly expressed in M-MDSCs isolated from pristane-induced through AMPK/mTOR signaling (132). LncRNA NEAT1 is closely related to the pathogenesis of immune-related diseases mediated by immune imbalance (144). LncRNA NEAT1 overexpressed in PMN-MDSCs of MRL/lpr mice, but not M-MDSCs, could significantly promote PMN-MDSCs to secrete BAFF and subsequently enhanced the activation of IFN-γ signaling in B cells, thereby promoting SLE to progress (134, 135). Further research showed MDSCs-derived Arg-1 promoted Th17 differentiation by elevating the amount of mmu-miR-322-5p in mice, which is homologous to hsa-miR-542-5p in humans. Furthermore, in humanized SLE mice, MDSCs-deplete group showed a declined proportion of Th17 in both PBMC and spleen, while the mRNAs expression levels of miR-542-5p and 3 polyamine synthetases were down-regulated in PBMC, spleen, and kidney. Overexpression of miR-542-5p, reversed the Th17 proportion and IL-17A expression and ROR&t in PBMC, spleen, and kidney in MDSCs-deplete group. Meanwhile, the positive relationship between polyamine/miR-542-5p and SLE disease progression through Th17 induction *in vitro*, which have also been fully verified *in vivo* in humanized mouse models. All data demonstrated that MDSCs-derived Arg-1 from SLE mice strongly promoted Th17 and Treg differentiation in a mmu-miR-322-5p–dependent manner, eventually elevating the Th17/Treg ratio and worsening SLE disease (133).

### Inflammatory bowel disease

Inflammatory bowel disease (IBD) is an idiopathic intestinal inflammatory disease involving ileum, rectum and colon (8). MDSCs have been shown to ameliorate inflammatory bowel disease in multiple mouse models of IBD. In the peripheral blood of IBD patients, CD14^+^HLA-DR^-/low^ cells are significantly increased and associated with disease activity (8). In a colitis mouse model induced by Helicobacter hepatica (HP), closely resembling human IBD, PMN-MDSCs and M-MDSCs in colon and spleen shown significantly accumulation in a time-dependent manner. Further study revealed that the recruitment of PMN-MDSCs was restricted and the inflammatory response mediated by MDSCs was inhibited when activating the I-cysteine/H2S pathway, thereby alleviating colonic inflammation (126). In the animal model of IBD induced by VILLAIN- hemagglutinin (HA), MDSCs accumulated in the spleen and intestine and only M-MDSCs prevented T cells to proliferate and induce T cells to apoptosis *via* cell-cell contact and NO-dependent manner (127). Similarly, in the dextran-sulfate sodium (DSS)-induced colitis mouse model, the frequency of MDSCs in the spleen was significantly increased and associated with symptoms of intestinal inflammation. Meanwhile, adoptive transfer of MDSCs reduced inflammation and promoted efficient colonic mucosal repair (128). Furthermore, PMN-Exo attenuated the severity of DSS-induced colitis *via* preventing inflammatory factor response produced by Th1 cells and expanding Treg cells in an Arg-1 activity-dependent manner (129). Hence, the accumulation of MDSCs in the site of inflammation may be effective to treat IBD. Although most studies *in vitro* demonstrated its anti-inflammatory effects by inhibiting the proliferation and function of T cells, MDSCs also contribute to chronic inflammation by promoting the expansion of effector T cells. In patients with IBD, PMN-MDSCs from the peripheral blood not only failed to suppress the autologous T cells response but also enhanced T cells proliferation *in vitro*, due to phenotypic switching and downregulation of CEBPβexpression in MDSCs under steady state. Meanwhile, these findings were consistently validated in mouse models (125).

Epigenetic mechanisms are also related to the pathogenesis of IBD. Enhancer of zeste homolog 2 (EZH2), acts as a major histone methyltransferase, facilitates the trimethylation of histone H3 on lysine 27 (H3K27) and silences target gene transcription. Recently, a report displayed that inhibition of EZH2 activity could improve the symptoms of experimental enteritis and delay the development of colitis-related cancers. Moreover, an increasing number of functional MDSCs was found in the colons in DSS-induced colitis. In addition, inhibition of EZH2 activity with GSK126, a selective EZH2 inhibitor, contributed to the differentiation of hematopoietic progenitor cells into MDSCs *in vitro*, thereby ameliorating colitis symptoms, whereas depletion of MDSCs exacerbated disease progression (130).

As discussed in this section, there are currently two different hypotheses regarding the function of MDSCs in autoimmune diseases, namely pro-inflammatory and anti-inflammatory theories. According to the above two articles, it is not difficult to conclude that MDSCs play a pro-inflammatory role by promoting T cell proliferation and increasing the number of Th17 cells, thus exacerbating the progression of autoimmune disease; while MDSCS play an anti-inflammatory role by inhibiting T cell proliferation and promoting the differentiation of Treg cells, thus alleviating the symptoms of autoimmune disease. However, about these two contradictory functions, the reason may be that the immune microenvironment can affect the development and function of MDSCs, M-MDSCs and PMN-MDSCs have different immunosuppressive functions in different stages of autoimmune disease, and regulate the immune response in different ways. In addition, another possible reason is the unreliable detection of MDSCs by surface markers. As described in the previous section, it is difficult to distinguish these cells from their precursors or from neutrophils and monocytes relying solely on surface markers. Therefore, measurements of transcription factors and immunomodulatory molecules, together with surface markers, are necessary for accurate identification of MDSCs subtypes. On the other hand, it has been found that the use of different proportions of MDSCs (as effector cells) versus T cells (as target cells) may induce different immune responses in cell culture-based studies, leading to this controversy. Data from the new study suggest that the *in vitro* regulatory function of MDSCs is entirely dependent on the frequency of these cells being used as effector cells. In summary, MDSCs have a strong potential to regulate immune imbalance occurring in autoimmune disorders, but the functional differences of MDSC subsets need to be further elucidated.

## Therapeutic potential of MDSCs on autoimmunity disease

Autoimmune diseases usually require prolonged treatment with immunosuppressive drugs. Considering that the long-term use of these drugs at high doses may cause many unexpected side effects and even an increased risk of life-threatening opportunistic infections and malignant tumors, therapeutic strategies targeting immunosuppressive cells show promising application prospects. The results of adoptive transfer of MDSCs in animal models of many autoimmune diseases lead us to believe that MDSCs have important therapeutic effects, although few studies have been conducted in humans. However, at the time of this review, clinical trials of adoptive MDSCs for autoimmune diseases had not been registered with clinicaltrials.gov or the European Union Clinical Trials Registry.

The conflicting evidence for the role of MDSCs in autoimmune diseases has been presented in the previous chapter, therefore, the effect of MDSCs-based cellular therapy in autoimmune diseases is also inconsistent. Ionnou et al. demonstrated that adoptive transfer of PMN-MDSCs derived from myelin antigen treated mice reduced spinal cord paralysis and inflammatory lesions compared to untreated controls (111). On the contrary, the study of Yi et al. confirmed that adoptively transferred MDSCs purified from EAE mice promoted the differentiation of Th17 cells and increased the level of IL-17 *in vitro* (136). The study of Mildner et al. demonstrated that adoptively transferred M-MDSCs are recruited to the inflammatory sites of the brain and thus contribute to maintain the inflammatory response (109). Similarly, there are conflicting data on the effects of adoptive transfer of MDSCs for the treatment of RA. Adoptively transferred MDSCs have been shown to differentiate into osteoclasts and contribute to bone resorption (145). Conversely, In multiple mouse models of RA, adoptive transfer of MDSCs reduced the relative ratio of Th1 and Th17 cells, accompanied by an increase in Treg numbers, and decreased serum inflammatory factors including TNF-α, IL-6, and IL-17, resulting in remission of clinical and histological manifestations of RA [118, 119, 146]. Furthermore, in addition to using whole intact cells, adoptive transfer of MDSC-derived exosomes has also been shown to improve symptoms of RA in mice (131). PMN-MDSC derived endosome (PMN-Exo) treatment decreased the populations of Th1 and Th17 cells. miRNAs isolated from PMN-Exo have been shown to affect T cell populations by down-regulating the expression of T-bet and STAT3, respectively (131). Similarly, the role of adoptive transfer of MDSCs in SLE varies. In a humanized mouse model of SLE, adoptive transfer of MDSCs from human SLE patients promoted IL-17 production, accompanied by nephritis and anti-dsDNA antibodies in mice, leading to increased clinical symptoms (8). Conversely, some studies have shown attenuation of clinical manifestations of SLE *via* the adoptive transfer of MDSCs (123, 124). In a pristane-induced lupus model, the adoptive transfer of M-MDSCs reduced the production of antibody and inhibited the proliferation of T cells in an iNOS and PGE dependent manner. Additionally, changes in the T cell population were also observed, with fewer Th1 cells and more Treg cells. In a sanroque mutation-driven model, the adoptive transfer of MDSCs derived from healthy mice decreased inflammatory cell infiltration in the liver and spleen, and reduced anti-dsDNA antibodies and proteinuria in the model mice, thereby alleviating clinical symptoms. Meanwhile, analysis of lymphocytes isolated from this model showed that MDSCs treated mice had increased IL-10^+^ B cells and decreased Th1, Th17 and Tfh cells. MDSCs have also been shown to be associated with the development and progression of inflammatory bowel disease. In multiple models of IBD mice, adoptive transfer of MDSCs from spleen cells of colitis mice reduced intestinal immune infiltration and the concentrations of inflammatory cytokines such as TNFα, IFN-γ, and IL-17 in colon tissue, thereby alleviating mucosal destruction of intestinal tissue and improving clinical symptoms in IBD mice.

In conclusion, MDSC-based cell therapy has shown promising clinical application in preclinical animal models. However, mixed results from animal models, MDSCs subtypes, and differences among researchers highlight the nuances of this treatment. At the same time, therapies targeting the epigenetic modification mechanisms of MDSCs have been well studied and achieved clinical application in the treatment of tumors, but little research has been done in the treatment of autoimmune diseases. Therefore, the functional diversity and therapeutic potential of MDSCs autoimmune diseases can be further explained from the perspective of epigenetic modification mechanism.

## Conclusion

MDSCs are a group of highly heterogeneous cell subsets with a variety of phenotypic characteristics, and their differentiation, expansion and activation *in vivo* are highly dependent on immune micro. MDSCs with different phenotypic characteristics regulate immune effector cells through different mechanisms, thus affecting immune response. In addition, the differentiation, expansion and activation of MDSCs are also regulated by a variety of epigenetic factors, especially the non-coding RNAs (20). In certain pathological conditions, MDSCs act as a double-edged sword, either favor disease outcome or exacerbate disease progression. By reviewing these two types of literature, many papers supporting the inflammation theory argue that MDSCs promotes T cells proliferation and increases the number of Th17 cells, eventually leading to immune imbalance, while other papers focus on the anti-inflammatory theory, suggesting that MDSCs increases the number of Treg cells and Breg cells, thus maintaining immune tolerance (146). The heterogeneity of MDSCs, the plasticity of microenvironment on MDSCs, and the limitation of detection methods are all possible reasons for this contradictory result. In addition, the epigenetic mechanisms affecting the role of MDSCs to regulate the pathogenesis of inflammatory autoimmune disease have been well studied, especially the non-coding RNAs regulation. MDSCs-related miRNAs can be used not only as monitoring indicators of MDSC activity, but also as potential biomarkers for predicting autoimmune disease progression. Finally, the latest development of MDSCs-based cell therapy is also summarized in the present review. Although there are still many theoretical mechanistic and technical methodological challenges to MDSCs-based cell therapy, we believe that these challenges will eventually be overcome and the cell therapy of autoimmune diseases based on MDSCs will make significant progress and gain clinical application in the future.

## Author contributions

DX and CL contributed to the conception, and wrote the manuscript. YX and MH designed the Figures and Tables. DC and JX conceived the topic and revised the manuscript. All authors contributed to the article and approved the submitted version.
